# BnaMPK3s promote organ size by interacting with BnaARF2s in *Brassica napus*

**DOI:** 10.1111/pbi.14013

**Published:** 2023-02-02

**Authors:** Xia Tian, Xiwen Yu, Zhijie Wang, Liang Guo, Jinxing Tu, Jinxiong Shen, Bin Yi, Tingdong Fu, Jing Wen, Chaozhi Ma, Cheng Dai

**Affiliations:** ^1^ National Key Laboratory of Crop Genetic Improvement Huazhong Agricultural University Wuhan China; ^2^ Hubei Hongshan Laboratory Wuhan China; ^3^ Huaiyin Institute of Agricultural Sciences of Xuhuai Region in Jiangsu, Huai'an Key Laboratory for Agricultural Biotechnology Huai'an China; ^4^ College of Plant Science and Technology Huazhong Agricultural University Wuhan China

**Keywords:** *Brassica napus*, *BnaMPK3*, *BnaARF2*, organ size

The larger organs have potential economic value in *Brassica napus* improvement and bioenergy production. Mitogen‐activated protein kinase (MAPK) cascades are highly conserved signalling modules, playing pivotal roles in multiple processes related to plant growth and development (Xu and Zhang, 2015). We identified two homologues of *AtMPK3* in *B. napus*, *BnaA06.MPK3* (*BnaA06g18440D*) and *BnaC03.MPK3* (*Bna03g55440D*), respectively (Figure S1a,b,e). These two *BnaMPK3s* were highly expressed in vegetative tissues and increasingly expressed in seeds during development (Figure S1c,d). We found that the seed size of Arabidopsis *mpk3* mutants was significantly smaller than wild type (Figure S2). To examine the roles of *BnaMPK3s* in the seed size control, the *BnaMPK3s* double mutants (*bnampk3* ^*CR*^, L4 and L5) were generated by CRISPR/Cas9 toolkit in T_0_ generation and further confirmed in T_1_ generation (Figure S3; Table S1). The cotyledon size and fresh weight of T_1_ *bnampk3* ^*CR*^ plants were less than WT (Figure S4a–d). The silique width, 1000 seed weight (TSW) and the yield per plant were decreased, while other yield related agronomic traits (e.g. branch number) were not affected (Figure 1a, Figure S4e), suggesting MPK3s play conserved functions in promoting organ size.

**Figure 1:**
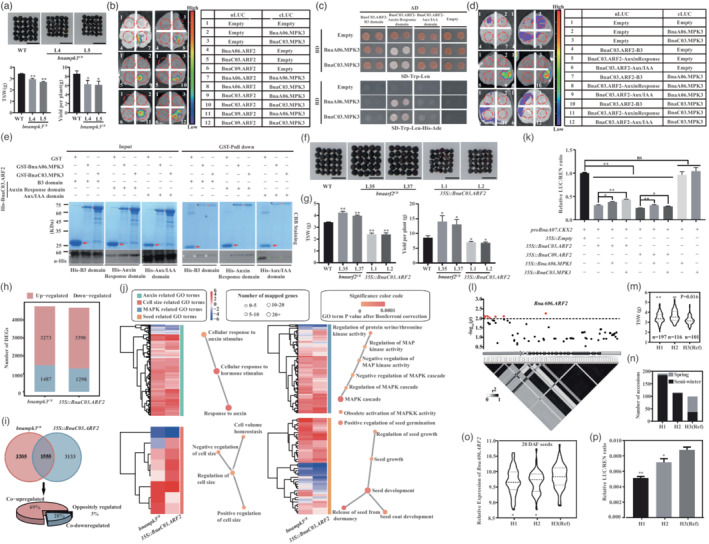
BnaMPK3s positively regulate organ size via interacting with BnaARF2s in *Brassica napus*. (a) The image of seed size, quantitation of 1000 seed weight and yield per plant of *bnampk3* ^*CR*^ and WT. Bar = 5 mm. (b) BnaMPK3s interacted with BnaARF2s in split luciferase complementary assay. (c)–(e) BnaMPK3s interacted with BnaC03.ARF2 Auxin Response domain in yeast‐two‐hybrid (c), split luciferase complementary (d) and pull‐down assay (e). In (e), GST, GST‐BnaA06.MPK3 and GST‐BnaC03.MPK3 were labelled by arrow, black and red asterisk, respectively; the His‐BnaC03.ARF2‐B3, His‐BnaC03.ARF2‐Auxin Response and His‐BnaC03.ARF2‐Aux/IAA were labelled by white, blue and green asterisk, respectively. (f), (g) The image (f) and bar graph (g) showed the seed size, 1000 seed weight and yield per plant of WT, *bnaarf2* ^*CR*^ and *35 S::BnaC03.ARF2*. Bar = 5 mm. (h) The number of DEGs in *bnampk3* ^*CR*^ and *35 S::BnaC03.ARF2* compared with WT. (i) The co‐regulated DEGs by BnaMPK3 and BnaARF2. (j) GO terms of co‐up‐regulated genes in *bnampk3* ^*CR*^ and *35 S::BnaC03.ARF2*. (k) The LUC/REN ratios of *pBnaA07.CKX2‐LUC* expression in dual‐luciferase assay. (l) Manhattan plot and LD statistic *r* ^2^ for *BnaA06.ARF2* (promoter and gene body). The grey arrow, grey rectangles, solid lines and black rectangles represented the promoter, UTRs, introns and exons, respectively. The red dot above the dotted line (significant threshold −log_10_(*p*) = −log_10_(0.01) = 2.0) represents a significantly associated SNP. The colour key (white to black) represents linkage disequilibrium values (*r* ^2^). (m) The distribution of 1000 seed weight in three haplotypes. The bars within violin plots represented 25th percentiles, medians and 75th percentiles. (n) The haplotype distribution in different *B. napus* ecotypes. (o) The relative expression levels of *BnaA06.ARF2* in different haplotypes at the 20‐DAF seeds. (p) The relative LUC activity (LUC/REN) from the transient expression of *LUC* driven by the promoters of three haplotypes. In (a), (g), (k), (m), (o) and (p), data were analysed by Student's *t*‐test (*P < 0.05; **P < 0.01). WT: Westar. L4 and L5: *bnampk3* ^*CR*^, L35 and L37: *bnaarf2* ^*CR*^, and L1 and L2: *35 S::BnaC03.ARF2*.

MAPKs regulate diverse biological processes through interacting with different proteins (Guan *et al*., 2014). Thus, we used BnaC03.MPK3 as bait to screen the *B. napus* cDNA library, and identified BnaC03.ARF2 (506–1536 bp) that could interact with BnaC03.MPK3. There were four homologues of *ARF2* in *B. napus*, which were widely expressed in different tissues (Figure S5a,b). All *BnaARF2s* were down‐regulated during the seed development (Figure S5c). Then, the interaction between MPK3 and ARF2 were confirmed by using *B. napus* and *Arabidopsis* MPK3 and ARF2 homologues (Figure 1b; Figures S6 and S7). Further analysis revealed that BnaMPK3s interacted with BnaARF2s via Auxin Response domain (Figure 1c–e; Figure S8).

The seed size was enlarged in Arabidopsis *atarf2* ^*CR*^ mutants (Figure S9), consistent with previous report (Schruff *et al*., 2006). Two *BnaARF2* quadruple mutants (*bnaarf2* ^*CR*^, L35 and L37, T_2_ generation, Table S2) were isolated from our previous study (Tang *et al*., 2018), which also had enlarged organs (Figure S10a–d). Specifically, silique length and width, 1000 seed weight, and yield per plant of *bnaarf2* ^*CR*^ were all greatly increased (Figure 1f,g, Figure S10e). On the contrary, overexpressing *BnaC03.ARF2* reduced the organ size, especially the TSW and yield per plant were lower than WT (Figure 1f,g, Figure S11). These results suggest that *BnaARF2*s negatively regulate organ size and seed weight. Furthermore, the seed size of *atmpk3* ^*CR*^ *atarf2* ^*CR*^ double mutant was similar to that of *atarf2* ^*CR*^ (Figure S12), indicating that *ARF2* is epistatic to *MPK3* in regulating seed size.

To identify the downstream genes of MPK3‐ARF2 module, RNA‐seq analysis was performed using 2‐week‐old seedlings of WT, *bnampk3* ^*CR*^ and *35 S::BnaC03.ARF2*. Compared with WT, a total of 4760 and 4688 differential expressed genes (DEGs) were identified in *bnampk3* ^*CR*^ and *35 S::BnaC03.ARF2*, respectively (Figure 1h; Table S3). Among them, 1555 genes were co‐regulated by *BnaMPK3* and *BnaARF2* (Figure 1i; Table S4). The co‐up‐regulated genes were mainly enriched in the GO terms of seed development, cell size, MAPK cascade and auxin response network (Figure 1j; Table S5). The homologue genes involved in seed size regulation, such as *BnaA07.CKX2* and *BnaA06.ABA2* (IKU pathway genes), *BnaA08.SK41* (Auxin signalling related gene), *BnaA05.BG1* (Auxin transporter related gene) and *BnaA05.IQD26* (BR related gene), were confirmed to have consistent expression changes in WT, *bnampk3* ^*CR*^, *bnaarf2* ^*CR*^ and *35 S::BnaC03.ARF2* seedlings and developing seeds (Figure S13), suggesting that these genes could be co‐regulated by *BnaMPK3* and *BnaARF2*. Furthermore, the dual‐luciferase assay showed that expression of *pBnaA07.CKX2‐LUC* was inhibited by BnaARF2s but derepressed by co‐infiltration of *BnaARF2s* and *BnaMPK3s* (Figure 1k, Figure S14). These results suggest that the BnaARF2s transcriptional activities are regulated by BnaMPK3s.

To validate the function of *BnaMPK3* and *BnaARF2* homologues in other *B. napus* accessions, the candidate gene association analysis of 1000 seed weight (TSW) was performed using 505 *B. napus* natural population (Tang *et al*., 2021). Only seven TSW associated SNPs (promoter: 6 SNPs; fifth exon: 1 SNP) were identified in *BnaA06.ARF2*, but not in *BnaMPK3s* and other *BnaARF2s* (Figure 1l; Figure S15; Table S6). The SNP in the fifth exon only caused a synonymous mutation (Table S6). Then, these SNPs were grouped into three distinct haplotypes, H1, H2 and H3 (reference sequence). TSW of the H1 and H2 haplotypes was significantly higher than the H3 haplotype (Figure 1m). Interestingly, H1 and H2 were enriched in semi‐winter type, whereas H3 was predominant in spring type of *B. napus* (Figure 1n), and TSW of spring *B. napus* was lower than semi‐winter type (Figure S16). In natural population, the expression of *BnaA06.ARF2* in H1 and H2 haplotypes were lower than that of H3 haplotype at 20‐DAF seeds (Figure 1o). Moreover, the relative LUC activities driven by H1 and H2 haplotype promoters were lower than that driven by H3 haplotype (Figure 1p), suggesting that the varied expression levels of *BnaA06.ARF2* is closely associated with the SNPs in its promoter.

In this study, several lines of evidence showed that the BnaMPK3s‐BnaARF2s module is critical for the regulation of organ size and yield in *B. napus*. The natural mutations in the promoter of *BnaA06.ARF2* may contribute to the TSW of *B. napus*. These findings provide valuable target genes for genome editing and molecular maker development that can be applied in *B. napus* breeding.

## Conflicts of interest

The authors declare no conflicts of interest.

## Author contribution

C.D. designed the research. X.T., X.Y and Z.W. performed the experiments and data analysis. L.G. provided the TWAS data. J.T., J.S., B.Y., T.F. and C.M. provided laboratory support. C.D. and X.T. wrote the manuscript. All authors read and approved the manuscript.

## Supporting information


**Figure S1** Characterization of MPK3 homologues in *B. napus*.
**Figure S2** Knockout of *AtMPK3* decreased seed size.
**Figure S3** The genotype of *bnampk3* ^*CR*^ in T_1_ generation.
**Figure S4** The agronomic traits of *bnampk3* ^*CR*^ and WT.
**Figure S5** Characterization of ARF2 homologues in *B. napus*.
**Figure S6** BnaMPK3s interacted with BnaARF2s in Y2H assay.
**Figure S7** AtMPK3 interacted with AtARF2.
**Figure S8** BnaMPK3s interacted with the Auxin Response domain of BnaARF2s.
**Figure S9** *atarf2* ^*CR*^ increased the seed size.
**Figure S10** The agronomic traits of *bnaarf2* ^*CR*^ and WT.
**Figure S11** The cotyledon and leaf size of *35 S::BnaC03.ARF2* and WT.
**Figure S12** ARF2 is epistatic to MPK3 in regulating seed size.
**Figure S13** The expression levels of selected DEGs in different materials.
**Figure S14** The constructs used for the dual‐luciferase assay.
**Figure S15** Association analysis the *BnaA06.MPK3*, *BnaARF2s*.
**Figure S16** The TSW of spring and semi‐winter cultivars.

**Table S1** Genotype of *bnampk3* ^*CR*^ double mutants.

**Table S2** Genotype of *bnaarf2* ^*CR*^ quadruple mutants.

**Table S3** List of DEGs in *bnampk3* ^*CR*^ and *35 S::BnaC03.ARF2* relative to WT.

**Table S4** The co‐regulated DEGs by *BnaMPK3* and *BnaARF2*.

**Table S5** GO enrichment analysis of co‐up‐regulated genes in *bnampk3* ^*CR*^ and *35 S::BnaC03.ARF2*.

**Table S6** Variations in *BnaA06.ARF2* promoter and genomic regions.

**Table S7** List of primer sequences used in this study.
