# Supplementary material for: BnaMPK3s promote organ size by interacting with BnaARF2s in Brassica napus
Source: Plant Biotechnol J. 2023 Feb 2;21(5):899–901. doi: 10.1111/pbi.14013 (PMC10106852; doi:10.1111/pbi.14013)
Supplement: Supplementary file 1 — Figure S1 Characterization of MPK3 homologues in B. napus. Figure S2 Knockout of AtMPK3 decreased seed size. Figure S3 The genotype of bnampk3 CR in T1 generation. Figure S4 The agronomic traits of bnampk3 CR and WT. Figure S5 Characterization of ARF2 homologues in B. napus. Figure S6 BnaMPK3s interacted with BnaARF2s in Y2H assay. Figure S7 AtMPK3 interacted with AtARF2. Figure S8 BnaMPK3s interacted with the Auxin Response domain of BnaARF2s. Figure S9 atarf2 CR increased the seed size. Figure S10 The agronomic traits of bnaarf2 CR and WT. Figure S11 The cotyledon and leaf size of 35 S::BnaC03.ARF2 and WT. Figure S12 ARF2 is epistatic to MPK3 in regulating seed size. Figure S13 The expression levels of selected DEGs in different materials. Figure S14 The constructs used for the dual‐luciferase assay. Figure S15 Association analysis the BnaA06.MPK3, BnaARF2s. Figure S16 The TSW of spring and semi‐winter cultivars. [file PBI-21-899-s007.docx]

**Supplemental Information**

**BnaMPK3s promote organ size by interacting with BnaARF2s in *Brassica napus***

Xia Tian^1,2^, Xiwen Yu^1,3^, Zhijie Wang^1,2^, Liang Guo^1,2^, Chaozhi Ma^1,2^ and Cheng Dai^1,2^

^1.^National Key Laboratory of Crop Genetic Improvement, Huazhong Agricultural University, Wuhan 430070, China

^2.^ Hubei Hongshan Laboratory, Wuhan, China

^3.^ Huaiyin Institute of Agricultural Sciences of Xuhuai Region in Jiangsu, Huai’an Key Laboratory for Agricultural Biotechnology, Huai’an, China

Running title: The roles of *BnaMPK3* in organ size

The author responsible for the distribution of materials integral to the findings presented in this article in accordance with the policy described in the Instructions for Authors is:

Cheng Dai ([cdai@mail.hzau.edu.cn](mailto:cdai@mail.hzau.edu.cn)).

**# For whom correspondence should be addressed.**

Dr. Cheng Dai, Email: [cdai@mail.hzau.edu.cn](mailto:cdai@mail.hzau.edu.cn)

National Key Laboratory of Crop Genetic Improvement, Huazhong Agricultural University, Wuhan 430070, China

**Contents**

**Supplemental Materials and Methods.****Figure S1.** Characterization of MPK3 homologues in *B. napus*.

**Figure S2.** Knockout of *AtMPK3* decreased seed size.

**Figure S3.** The genotype of *bnampk3^CR^* in T_1_ generation.

**Figure S4.** The agronomic traits of *bnampk3^CR^* and WT.

**Figure S5.** Characterization of ARF2 homologues in *B. napus*.

**Figure S6.** BnaMPK3s interacted with BnaARF2s in Y2H assay.

**Figure S7.** AtMPK3 interacted with AtARF2.

**Figure S8.** BnaMPK3s interacted with the Auxin Response domain of BnaARF2s.

**Figure S9.** *atarf2^CR^* increased the seed size.

**Figure S10.** The agronomic traits of *bnaarf2^CR^* and WT.

**Figure S11.** The cotyledon and leaf size of *35S::BnaC03.ARF2* and WT.

**Figure S12.** ARF2 is epistatic to MPK3 in regulating seed size.

**Figure S13.** The expression levels of selected DEGs in different materials.

**Figure S14.** The constructs used for the dual-luciferase assay.

**Figure S15.** Association analysis the *BnaA06.MPK3*, *BnaARF2s*.

**Figure S16.** The TSW of spring and semi-winter cultivars.

**Table S1.** Genotype of *bnampk3^CR^* double mutants.

**Table S2.** Genotype of *bnaarf2^CR^* quadruple mutants.

**Table S3.** List of DEGs in *bnampk3^CR^* and *35S::BnaC03.ARF2* relative to WT.

**Table S4.** The co-regulated DEGs by *BnaMPK3* and *BnaARF2*.

**Table S5.** GO enrichment analysis of co-upregulated genes in *bnampk3^CR^* and *35S::BnaC03.ARF2*.

**Table S6.** Variations in *BnaA06.ARF2* promoter and genomic regions.

**Table S7.** List of primer sequences used in this study.

**Supplemental Materials and Methods**

**Plant materials and growth conditions**

The *Brassica napus* mutants of *BnaMPK3* were generated by the CRISPR/Cas9 technology in *B. napus* (Westar). The quadruple mutants of *BnaARF2s* (L35 and L37) were obtained from previous study (Tang et al. 2018). All *Brassica napus* mutants, *BnaC03.ARF2* overexpression transgenic lines and wild-type (WT, Westar) seeds were soaked in water for 7 days and then placed in Hoagland media or soil to analysis the seedling phenotype. All plants were cultivated in a growth room under a light intensity of 100 μmol m^−2^ s^−1^ with a 16/8 h light/dark photoperiod at 22°C. To analysis the agronomic traits in the field, all mutants, *BnaC03.ARF2* overexpression transgenic lines and wild-type (WT, Westar) were planted in the experimental field at Huazhong Agricultural University, Wuhan, China.

The Arabidopsis *AtMPK3* T-DNA insertion mutant, CS331909, was obtained from the Nottingham Arabidopsis Stock Centre (NASC). The mutants of *AtMPK3* and *AtARF2* were generated by the CRISPR/Cas9 technology in *Arabidopsis thaliana* (Col). The double mutant *atmpk3^CR^atarf2^CR^* were generated by crossing *atmpk3^CR^* and *atarf2^CR^*. Seeds were vernalized for 2 days at 4°C, and germinated on MS media for seven days. The Arabidopsis seedlings were further grown in a greenhouse at a temperature of 22°C (day) and 18°C (night), 70% humidity.

**Plasmid construction**

The *GENE-sgRNA* plant expression vectors were constructed as previously described with minor modifications (Xing et al. 2014). The target sgRNA sequences were designed using the web server CRISPR-P (<http://cbi.hzau.edu.cn/cgi-bin/CRISPR>) (Lei et al. 2014). For *BnaMPK3s* double mutants, the sgRNAs were targeted the conserved region. Using *pCBC-DT1T2* as the template, two *AtU6 promoter-sgRNA-AtU6 terminator* cassettes were amplified by PCR using the primers listed in Table S7. Then the PCR fragments were inserted into *pKSE401G* by Golden Gate Assembly, and confirmed by Sanger sequencing. The construct information is from previous report (Yang et al. 2017).

The CDS of each gene was amplified from *B. napus* or *A. thaliana* cDNA by PCR using gene-specific primers, respectively. For the yeast two hybrid assay, the *BnaA06.ARF2*, *BnaC03.ARF2*, *BnaC09.ARF2* or *AtARF2* coding region was cloned into the vector *pGBKT7* as the bait (Clontech, USA), while the *BnaA06.MPK3*, *BnaC03.MPK3* or *AtMPK3* coding region was cloned into the vector *pGADT7* as the prey. For the split LUC assay, the CDS of *BnaARF2s* or *AtARF2* were subcloned into the vector *JW771* (*35S nLUC*) fused with the N terminus of LUC, and *BnaMPK3s* or *AtMPK3* were subcloned into the vector *JW772* (*35S cLUC*) fused with the C terminus of LUC (Chen et al. 2008). For the pull-down assay, the *BnaA06.MPK3* or *BnaC03.MPK3* coding region was cloned into the vector *pGEX-4T-3*. For the dual-luciferase assay, the reporter construct contained the firefly luciferase driven by *BnaA07.CKX2* promoter, and the Renilla luciferase (REN) was driven by the *CaMV 35S* promoter. The effector constructs contained *BnaMPK3s* or *BnaARF2s* driven by the *CaMV 35S* promoter. For the dual-luciferase assay, the reporter construct contained the firefly luciferase driven by different *BnaA06.ARF2* promoter. All primers are listed in Table S7.

To amplify the protein domain of BnaARF2s, the domain was first predicted by NCBI website (<https://www.ncbi.nlm.nih.gov/Structure/bwrpsb/bwrpsb.cgi>). Then, B3 domain, Auxin Response domain and Aux/IAA domain of three BnaARF2 homologs (BnaA06.ARF2: B3 domain (1-285 AA), Auxin Response domain (286-612 AA) and Aux/IAA domain (613-851 AA); BnaC03.ARF2: B3 domain (1-284 AA), Auxin Response domain (285-611 AA) and Aux/IAA domain (612-850 AA); BnaC09.ARF2: B3 domain (1-261 AA), Auxin Response domain (262-586 AA) and Aux/IAA domain (587-828 AA)) were amplified by PCR using specific primers. The relative fragments were subcloned into *pGADT7*, *JW771* (*35S nLUC*) and pET32a for yeast-two-hybrid, split luciferase assay, and protein expressed, respectively. All primers are listed in Table S7.

**Identification of transgenic plants**

To analyze the mutations caused by CRISPR/Cas9, genomic DNA was extracted from each transgenic plant using the CTAB method (Molecular Cloning, 3rd edition). The flanking sequence around the CRISPR target sites of *BnaARF2* transgenic plants (T_1_ and T_2_ generation) or *BnaMPK3* transgenic plants (T_0_ generation) were amplified by PCR using gene-specific primers. First, each PCR amplicon generated from WT genomic DNA was sub-cloned into the *pGEM-T* easy vector (A3600, Promega, USA) to confirm the primer specificity by Sanger sequencing. Then, most of the amplicons were directly sequenced to analyze the mutations. For the complex mutations, the amplicons were first sub-cloned into the *pGEM-T* easy vector, and about 10 clones of each amplicon were individually sequenced by Sanger sequencing.

The targeted mutations of AtMPK3(T_0_-T_2_), AtARF2(T_0_-T_2_), *BnaARF2* (T_2_ and T_3_ generation) and *BnaMPK3* (T_1_ generation) were further determined by using the Hi‐TOM platform (Liu et al. 2019). Target‐specific and barcoding PCR were performed to amplify the genomic region encompassing the specific targets of independent samples, and the resulting PCR products were mixed in equal amounts and purified for next‐generation sequencing (Novogene Bioinformatics Institute, China). The resulting sequencing data were then decoded by a corresponding online tool to track the mutations of the target sites (<http://www.hi-tom.net/hi-tom/>). The target‐specific primer sets are listed in Table S7.

To genotype the CS331909 T-DNA insertion mutant, primers RP (5′-GTGGTGGTGGAACAACATCTC-3′) and left border primer LB (5′-ATTTTGCCGATTTCGGAAC-3′) were used to identify the T-DNA insertion in the AtMPK3 gene. Primers LP (5′-AACCATCGTAAACCACACATG -3′) and RP (5′-GTGGTGGTGGAACAACATCTC-3′) were used to identify the wild-type AtMPK3 allele in the insertion line.

**Yeast two hybrid assays**

The yeast Gal4 system was employed for two-hybrid analysis of ARF2s and MPK3 protein interactions following yeast transformation handbook (Yeast Transformation System 2; Clontech, USA). To assess protein-protein interactions, the transformed yeast cells were re-suspended in ddH_2_O to an optical density at OD_600_ of 1.0. Samples (5 µL) of suspended yeast cells were spread on plates containing SD medium lacking Ade, His, Leu and Trp (SD-4) or SD medium lacking His, Leu and Trp (SD-3). To detect protein-protein interactions, plates were examined after 2 days of incubation at 30°C. In each experiment, a total of six clones were selected and tested.

**Split LUC complementation assay**

The split LUC complementation assay was performed as previously described (Duan et al. 2014). In brief, Agrobacterium tumefaciens (strain GV3101) cells containing the desired constructs were co-infiltrated into 4 to 5-week-old *N. benthamiana* leaves with infiltration buffer (10 mM MES, pH=5.8, 10 mM MgCl_2_ and 150 μM acetosyringone). The final concentrations of the bacteria were adjusted to an OD_600_ = 0.6 for each construct. Infected leaves were analyzed at 48h after infiltration. A low-light cooled CCD imaging apparatus (Lumazone Pylon 2048 B) was used to capture the LUC image.

**Protein expression and purification**

GST-BnaMPK3s plasmids were transformed into Escherichia coli BL21 cells and incubated overnight to induce expression of recombinant proteins in the presence of 0.1mM IPTG at 16 °C. The purification of GST fusion proteins was performed by Mag-Beads GST Fusion Protein Purification (BBI Life Science NO. C650031) and stored in aliquots at −80 °C. Truncated His-BnaARF2s plasmids were transformed into Escherichia coli BL21 cells and incubated overnight to induce expression of recombinant proteins in the presence of 0.1mM IPTG at 20 °C. The His fusion proteins were further purified using the Mag-Beads His-Tag protein purification (BBI Life Science NO. C650033) and stored in aliquots at −80 °C.

**Pull-down assay**

For pull-down assay, the purified equal volumes of GST or GST-BnaMPK3s were incubated with Glutathione Magarose Beads (BBI Life Science NO. C650031) at 25°C for 30min. After incubation, truncated His-BnaARF2 fusion protein was added to the target protein magnetic bead complex, and it was incubated for another 30min at 25 °C. The bead-bound proteins were washed with GST-wash buffer (BBI Life Science NO. C600326) five times. After washing, the bound proteins were eluted and separated using SDS-PAGE, and then detected with anti-His (D191001-0100, BBI) antibodies.

**Dual-luciferase assay**

For dual-luciferase assay, the effector and reporter constructs were first transformed into *Arabidopsis* mesophyll protoplasts as previously described (Huang et al. 2015) Then, dual-luciferase assay was followed the instruction of Dual-Luciferase® Reporter Assay System (E1910, Promega, WI, USA). The data were represented as the ratio of firefly to Renilla luciferase activity (LUC/Ren). Each data point consisted of at least three replicates, and three independent experiments were performed for each assay.

**RNA extraction and quantitative PCR analysis**

For tissue expression pattern, the tissue samples from root, stem, flower, flower bud, cotyledon, seeds at different development stage (10, 20, 30 days after pollination). were collected, and were frozen immediately by liquid nitrogen. Total RNA was extracted using a Plant Total RNA Isolation Kit (Tiangen, China) following the manufacturer’s instructions. Approximately 1.5 μg of total RNA was used for cDNA synthesis using Revert Aid first-strand cDNA Synthesis Kit (Thermo, Germany), and the qPCR reaction was followed previous report (Duan et al. 2020). All analyses were repeated three times using biological replicates. The *Bnaactin* (*BnaGAPDH, BnaC05g12400D*) gene served as the internal control. All primers are listed in Table S7.

**RNA-sequencing and data analysis**

For global transcript analysis, we harvested 1-week-old WT, *bnampk3^CR^* and *35S::BnaC03.ARF2* seedlings grown in water. Three samples were collected for each genotype and treatment and total RNA was extracted as described above. Construction of Illumina libraries and sequencing were completed by GENOSEQ. Totally, 270,853,307 high quality raw reads from 9 samples were generated by Illumina sequencing platform. SRA files for each sequencing were converted to the FASTQ format using fastq-dump (version 2.9.6) from the sratoolkit. Paied-end sequenced reads were quality-tested using FastQC (version 0.11.9). Before mapping to the reference genome, all raw reads were trimmed using fastp (version 0.22.0). Subsequently, all filtered clean reads were mapped to the *B. napus* reference genome (*Brassica_napus*_v4.1.chromosomes;https://www.genoscope.cns.fr/brassicanapus/data/) by hisat2 (version 2.2.1) with default parameters. Alignment sam files were then transformed to bam files by samtools (version 1.8) (Huang et al. 2009). Reads count numbers were calculated by featureCounts (version 2.0.0) (Liao et al. 2014). Different expression genes were analyzed by edgeR, an R based packages available from Bioconductor, using the former results of featureCounts. Then transcript abundance was measured as a Fold Change (FC) value using R script. Expression levels log_2_FC of genes were visualized by heatmap use pheatmap package. Functional annotations of GO terms in gene lists using TBtools (version 1.076) (Chen et al. 2020). Functional annotations were performed using GO molecular function, biological process, and cellular component annotation data set. Fisher’s Exact with FDR multiple test correction was used to compare enrichment to the genome distribution.

**Morphological analysis**

Average seed weight was weighted by an electronic analytical balance (BS223S; Sartorius, Gottingen, Germany) with mature dry seeds in batches of 1000. The seeds, and leaves were photographed using a digital camera (Canon EOS50D), and then, seed size, and leaf area were measured using Image J software. The seedlings for analysis were planted in an incubator. The samples for analysis of yield per plant, silique length, seed number per silique, plant height, primary branch number and seed oil content were all collected from the mature plants. The experiment was repeated three times. For each repeat, at least five plants of each genotype were used.

**Association analysis of BnaMPK3 and BnaARF2**

The association analysis was followed as previous reports (Wang et al. 2017). A total of 505 *B. napus* accessions collected from different research institutions were grown in Wuhan for two growth seasons (2012-2013, and 2013-2014). Field experiments were designed in a randomized complete block design with three replicates and plot size of 3 m^2^. The open‐pollinated seeds were harvested from 10 individual plants of each plot when they were mature and measured for TSW. An R script (www.eXtension.org/pages/61006) based on a linear model was used to obtain the best linear unbiased prediction of TSW as phenotypic values in each line.

The polymorphic SNPs of BnaMPK3 and BnaARF2 were genotyped by genomic DNA re‐sequencing (Tang et al. 2021). Association analysis was performed using the software Admixture (http://<https://speciationgenomics.github.io/ADMIXTURE/>) and Emmax (Kang et al. 2010). The LD statistic r^2^ was calculated using Haploview (Barrett et al. 2005).

**Sequence analysis of BnaARF2**

The AtARF2 protein sequence was used to do a blast search of the BnaARF2, BolARF2 and BraARF2 in the NCBI. The sequence alignment was performed using Clustal Omega (http://www.ebi.ac.uk/Tools/msa/clustalo). An unrooted phylogenetic tree was constructed using MEGA-X (http://www.megasoftware.net/) with the Maximum Likelihood statistical method and bootstrap analysis (1000 replicates).

**Sequence analysis of BnaMPK3**

The AtMPK3 protein sequence was used to do a blast search of the BnaMPK3 BolMPK3, BraMPK3, BniMPK3 and BjuMPK3 in the NCBI. An unrooted phylogenetic tree was constructed using MEGA-X (http://www.megasoftware.net/) with the Maximum Likelihood statistical method and bootstrap analysis (1000 replicates).

**Statistical analyses**

Statistical analysis was performed to identify significant between genotypes, using Student's t-test, at P values < 0.05 or <0. 01.

**Accession numbers**

Sequence data from this article can be found in the *Brassica napus*, *Arabidopsis thaliana*, *Brassica rapa*, *Brassica oleracea* and *Oryza* Genome database under the following accession numbers: *BnaA06.ARF2* (*BnaA06g21460D*), *BnaA09.ARF2* (*BnaA09g05840D*), *BnaC03.ARF2* (*BnaC03g52090D*), *BnaC09.ARF2* (*BnaC09g05450D*), *BnaA06.MPK3* (*BnaA06g18440D*), *BnaC03.MPK3* (*BnaC03g55440D*), *Bnaactin* (*BnaC05g12400D*), *AtMPK3 (AT3G45640)*, *AtARF2 (AT5G62000)*, *BraA06g020840.3C* (*BraMPK3*)*, BraA06g025480.3C*(*BraA06.ARF2*) *BraA09g007140.3C* (*BraA09.ARF2*), *BolMPK3* (*Bol041350*)，*BolC03.ARF2* (*Bol007816*), *BolC09.ARF2* (*Bol019214*) and *OsMPK3* (*Os06t0154500*).

**References for Materials and Methods**

Barrett JC, Fry B, Maller J, Daly MJ (2005) Haploview: analysis and visualization of LD and haplotype maps. Bioinformatics 21 (2):263-265. doi:10.1093/bioinformatics/bth457

Chen C, Chen H, Zhang Y, Thomas HR, Frank MH, He Y, Xia R (2020) TBtools: An Integrative Toolkit Developed for Interactive Analyses of Big Biological Data. Mol Plant 13 (8):1194-1202. doi:10.1016/j.molp.2020.06.009

Chen H, Zou Y, Shang Y, Lin H, Wang Y, Cai R, Tang X, Zhou JM (2008) Firefly luciferase complementation imaging assay for protein-protein interactions in plants. Plant Physiol 146 (2):368-376. doi:10.1104/pp.107.111740

Duan P, Rao Y, Zeng D, Yang Y, Xu R, Zhang B, Dong G, Qian Q, Li Y (2014) SMALL GRAIN 1, which encodes a mitogen-activated protein kinase kinase 4, influences grain size in rice. Plant J 77 (4):547-557. doi:10.1111/tpj.12405

Duan Z, Zhang Y, Tu J, Shen J, Yi B, Fu T, Dai C, Ma C (2020) The Brassica napus GATA transcription factor BnA5.ZML1 is a stigma compatibility factor. J Integr Plant Biol 62 (8):1112-1131. doi:10.1111/jipb.12916

Huang M, Hu Y, Liu X, Li Y, Hou X (2015) Arabidopsis LEAFY COTYLEDON1 Mediates Postembryonic Development via Interacting with PHYTOCHROME-INTERACTING FACTOR4. Plant Cell 27 (11):3099-3111. doi:10.1105/tpc.15.00750

Huang X, Qian Q, Liu Z, Sun H, He S, Luo D, Xia G, Chu C, Li J, Fu X (2009) Natural variation at the DEP1 locus enhances grain yield in rice. Nat Genet 41 (4):494-497. doi:10.1038/ng.352

Kang HM, Sul JH, Service SK, Zaitlen NA, Kong SY, Freimer NB, Sabatti C, Eskin E (2010) Variance component model to account for sample structure in genome-wide association studies. Nat Genet 42 (4):348-354. doi:10.1038/ng.548

Lei Y, Lu L, Liu HY, Li S, Xing F, Chen LL (2014) CRISPR-P: a web tool for synthetic single-guide RNA design of CRISPR-system in plants. Mol Plant 7 (9):1494-1496. doi:10.1093/mp/ssu044

Liao Y, Smyth GK, Shi W (2014) featureCounts: an efficient general purpose program for assigning sequence reads to genomic features. Bioinformatics 30 (7):923-930. doi:10.1093/bioinformatics/btt656

Liu Q, Wang C, Jiao X, Zhang H, Song L, Li Y, Gao C, Wang K (2019) Hi-TOM: a platform for high-throughput tracking of mutations induced by CRISPR/Cas systems. Sci China Life Sci 62 (1):1-7. doi:10.1007/s11427-018-9402-9

Tang S, Zhao H, Lu S, Yu L, Zhang G, Zhang Y, Yang QY, Zhou Y, Wang X, Ma W, Xie W, Guo L (2021) Genome- and transcriptome-wide association studies provide insights into the genetic basis of natural variation of seed oil content in Brassica napus. Mol Plant 14 (3):470-487. doi:10.1016/j.molp.2020.12.003

Tang T, Yu X, Yang H, Gao Q, Ji H, Wang Y, Yan G, Peng Y, Luo H, Liu K, Li X, Ma C, Kang C, Dai C (2018) Development and Validation of an Effective CRISPR/Cas9 Vector for Efficiently Isolating Positive Transformants and Transgene-Free Mutants in a Wide Range of Plant Species. Front Plant Sci 9:1533. doi:10.3389/fpls.2018.01533

Wang JL, Tang MQ, Chen S, Zheng XF, Mo HX, Li SJ, Wang Z, Zhu KM, Ding LN, Liu SY, Li YH, Tan XL (2017) Down-regulation of BnDA1, whose gene locus is associated with the seeds weight, improves the seeds weight and organ size in Brassica napus. Plant Biotechnol J 15 (8):1024-1033. doi:10.1111/pbi.12696

Xing HL, Dong L, Wang ZP, Zhang HY, Han CY, Liu B, Wang XC, Chen QJ (2014) A CRISPR/Cas9 toolkit for multiplex genome editing in plants. BMC Plant Biol 14:327. doi:10.1186/s12870-014-0327-y

Yang H, Wu JJ, Tang T, Liu KD, Dai C (2017) CRISPR/Cas9-mediated genome editing efficiently creates specific mutations at multiple loci using one sgRNA in Brassica napus. Sci Rep 7 (1):7489. doi:10.1038/s41598-017-07871-9

­­


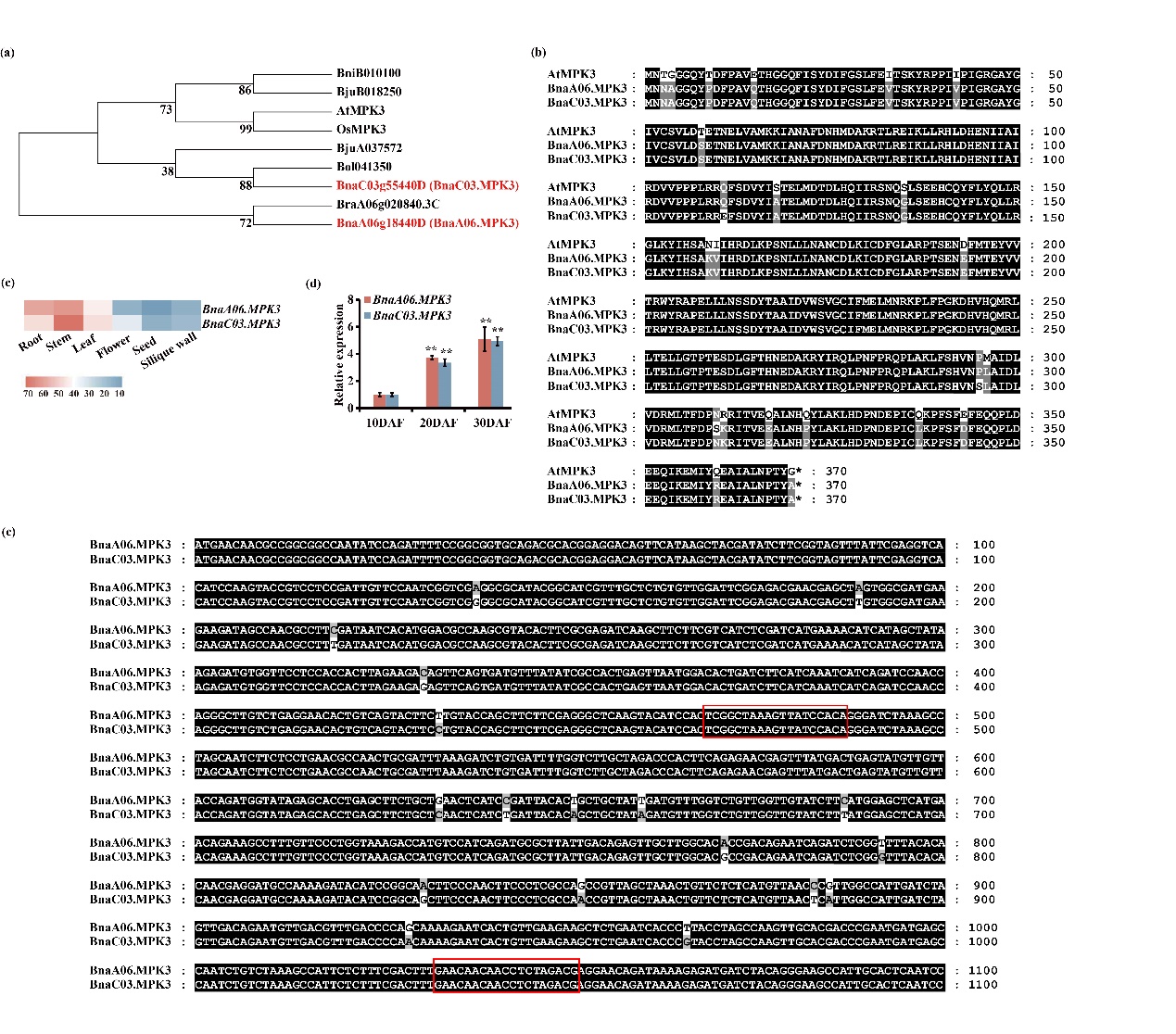


**Figure S1. Characterization of MPK3 homologues in *B. napus*.**

(a) Phylogenetic analysis of MPK3 homologs in *B. napus*, *B. rapa*, *B. oleracea* and *A. thaliana*. Two homologs of *BnaMPK3s* were marked with red. (b) Amino acid identities and similarities of MPK3s proteins in *A. thaliana* and *B. napus*. (c) Heatmap showed the RPKM values of *BnaMPK3s* in different tissues derived from the published RNA-seq dataset (https://www.ncbi.nlm.nih.gov/sra/). (d) Bar graph showed the expression level of *BnaMPK3s* in seeds after flowering. For each gene, the expression level of 10 DAF (day-after-flowering) was set as 1. *BnaActin* acted an internal control. Data were mean ± SE from three independent experiments, and analyzed by Student’s t-test (*, P < 0.05; **, P < 0.01). (e) Nucleotide identities and similarities of MPK3s in *B. napus*. The sgRNAs were denoted by red box.


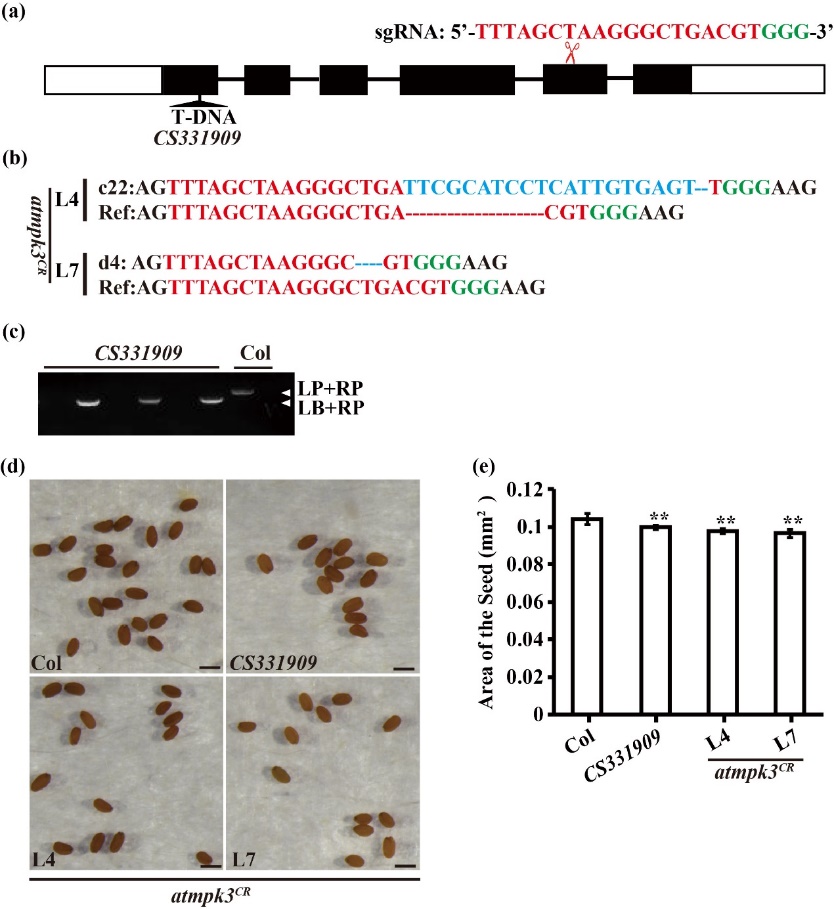


**Figure S2. Knockout of *AtMPK3* decreased seed size.**

(a) The *AtMPK3* gene model included six exons (box) separated by five introns (solid line). The black triangle indicated the T-DNA insertion site, and the red scissors in the gene model indicated the sgRNA target site. (b) Genotype of the *atmpk3^CR^* mutant in the T_1_ generation. The protospacer adjacent motif (PAM) region was marked in green. The sgRNA was denoted by red. The mutation sites were indicated with blue. c: complex (deletion and insertion); d: deletion. (c) Genotype of *atmpk3* T-DNA insertion mutant. The primer pairs LB/RP were used for confirming the T-DNA insertion, and the primer pairs LP/RP were used for identifying the homozygous mutant lines. (d)-(e) The image (d) and bar graph (e) showed the seed size of *atmpk3* and Col. Scale bars indicated 1 mm. In (d)-(e), *CS331909*: T-DNA insertion mutant; L4 and L7: two individual *atmpk3^CR^* lines; Col: wildtype. The data were analyzed by Student’s t-test (*, P < 0.05; **, P < 0.01).

**
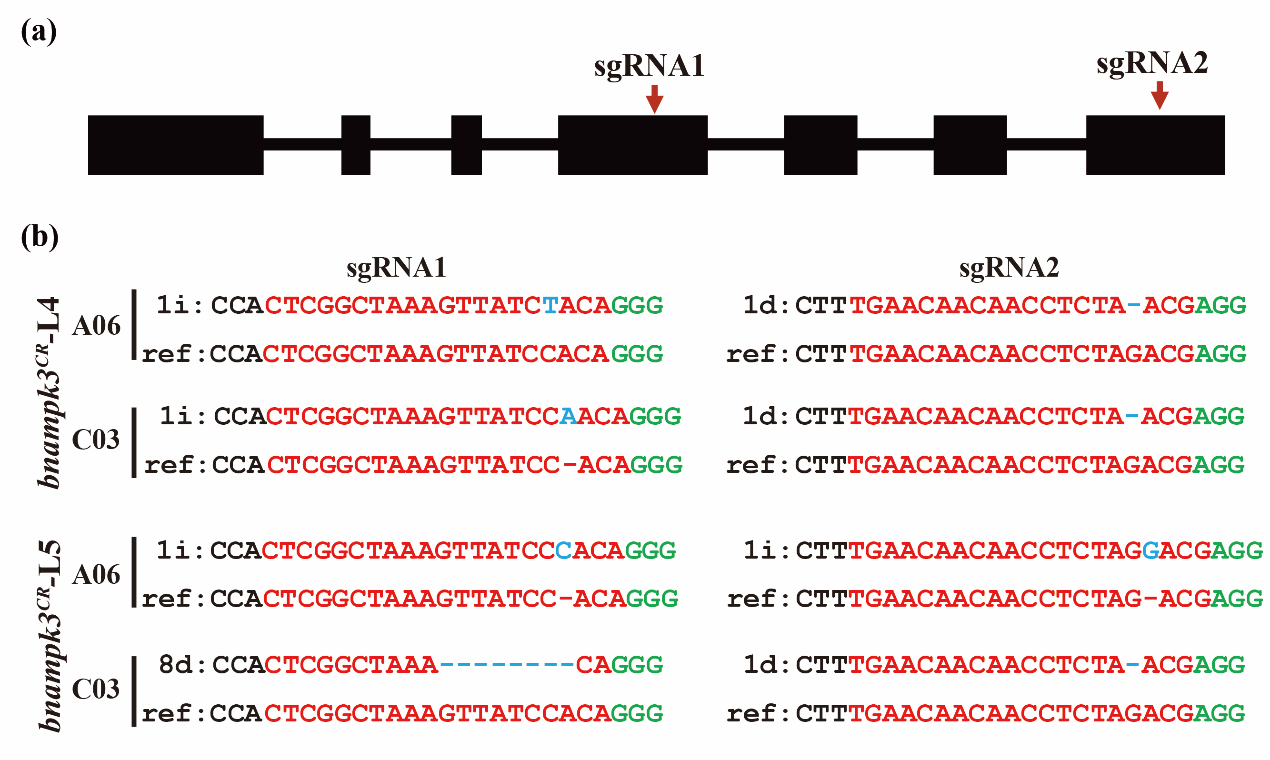
**

**Figure S3. The genotype of *bnampk3^CR^* in T_1_ generation.**

(a) The *BnaMPK3* gene model included seven exons (box) separated by six introns (solid line). The red arrows in the gene model indicated the sgRNA target site. (b) Genotype of the *bnampk3^CR^* mutant in the T_1_ generation. The protospacer adjacent motif (PAM) region was marked in green. The sgRNA was denoted by red. The mutation sites were indicated with blue. i: insertion; d: deletion.


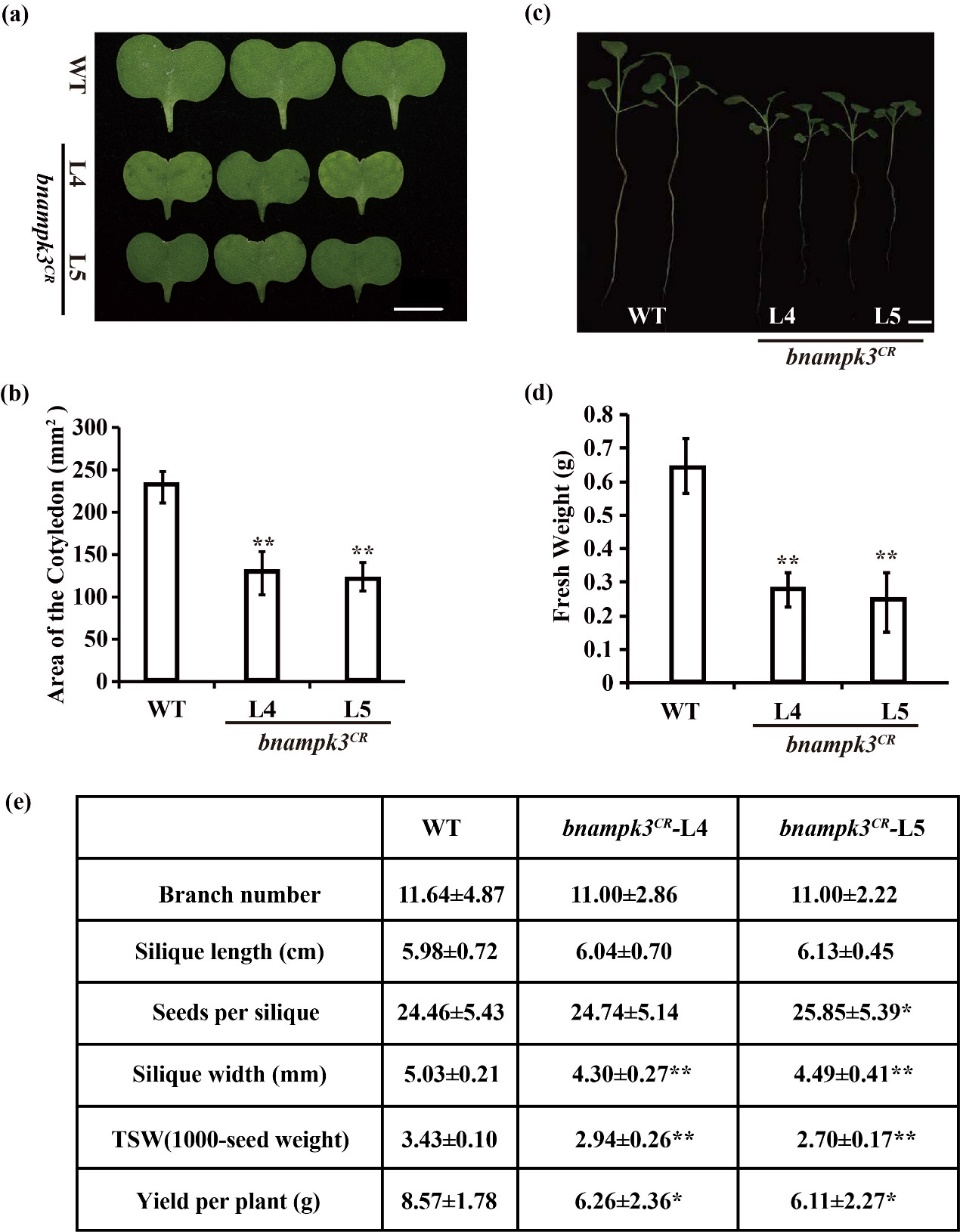


**Figure S4.** **The agronomic traits of *bnampk3^CR^* and WT.**

(a)-(b) The image (a) and bar graph (b) showed the cotyledon size of 14-day-old *bnampk3^CR^* and WT. In (a), scale bars indicated 10 mm. (c)-(d) The image (c) and bar graph (d) showed the fresh weight of 21-day-old *bnampk3^CR^* and WT. In (c), scale bars indicated 20 mm. (e) The table showed the yield related agronomic traits of *bnampk3^CR^* and WT. Data were analyzed by Student’s t-test (*, P < 0.05; **, P < 0.01).


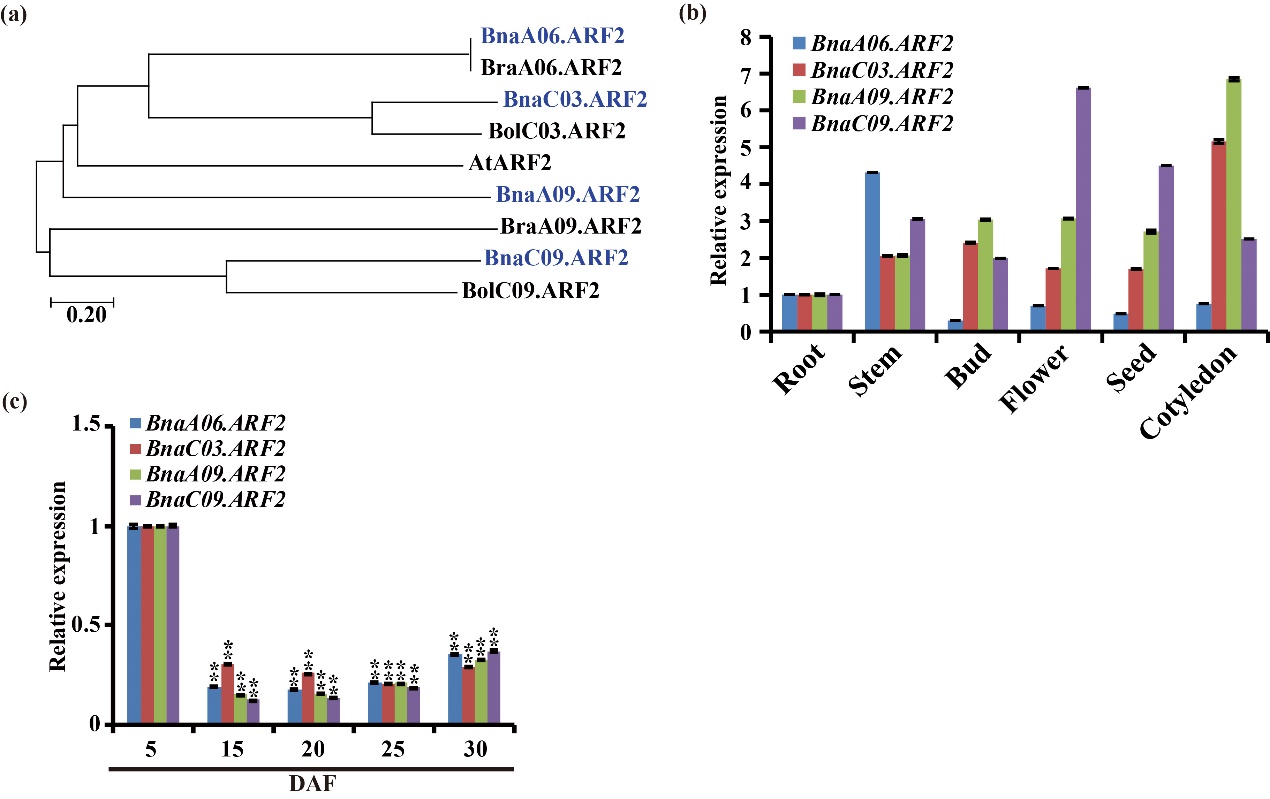


**Figure S5. Characterization of ARF2 homologues in *B. napus*.**

(a) Phylogenetic analysis of ARF2 in *B. napus*, *B. rapa*, *B. oleracea* and *A. thaliana*. Four homologs of BnaARF2 were marked with blue. (b) Bar graph showed the expression level of *BnaARF2*s in different tissues. For each gene, the expression level of root was set as 1. *BnaActin* acted an internal control. (c) Bar graph showed the expression level of *BnaARF2s* in seeds after flowering. For each gene, the expression level at 5 DAF was set as 1. *BnaActin* acted an internal control. In (b) and (c), Data were mean ± SE from three independent experiments, and analyzed by Student’s t-test (*, P < 0.05; **, P < 0.01).


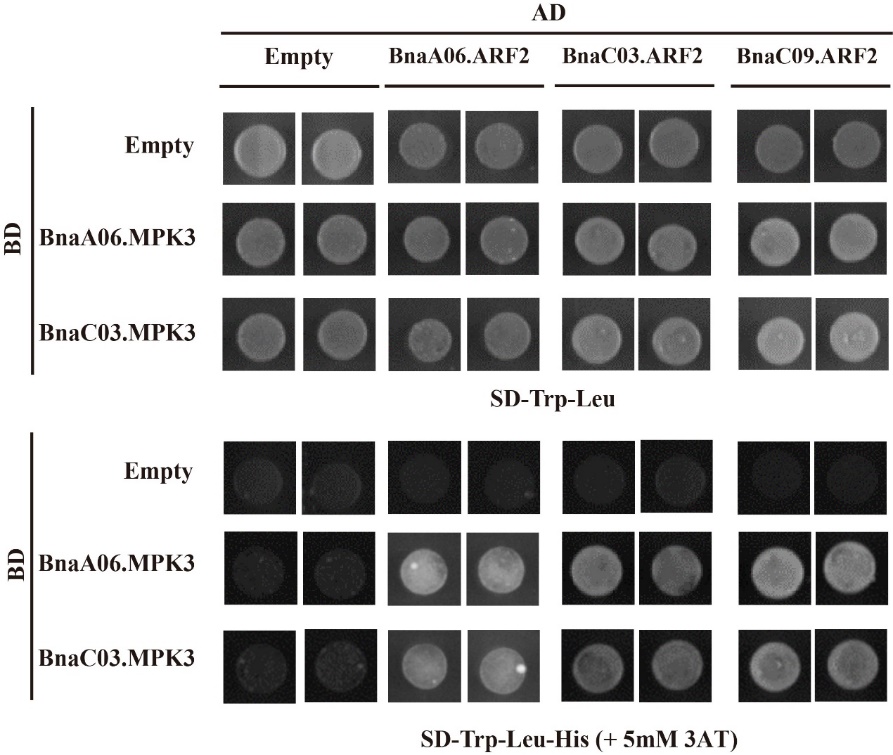


**Figure S6. BnaMPK3s interacted with BnaARF2s in Y2H assay.**

AD, GAL4 activation domain fusion; BD, GAL4-binding domain fusion. BD-Empty/AD-Empty was used as negative control. Yeast cells were cultured on SD/-Trp-Leu or SD/-Trp-Leu-His (+ 0.5 mM 3AT) media.


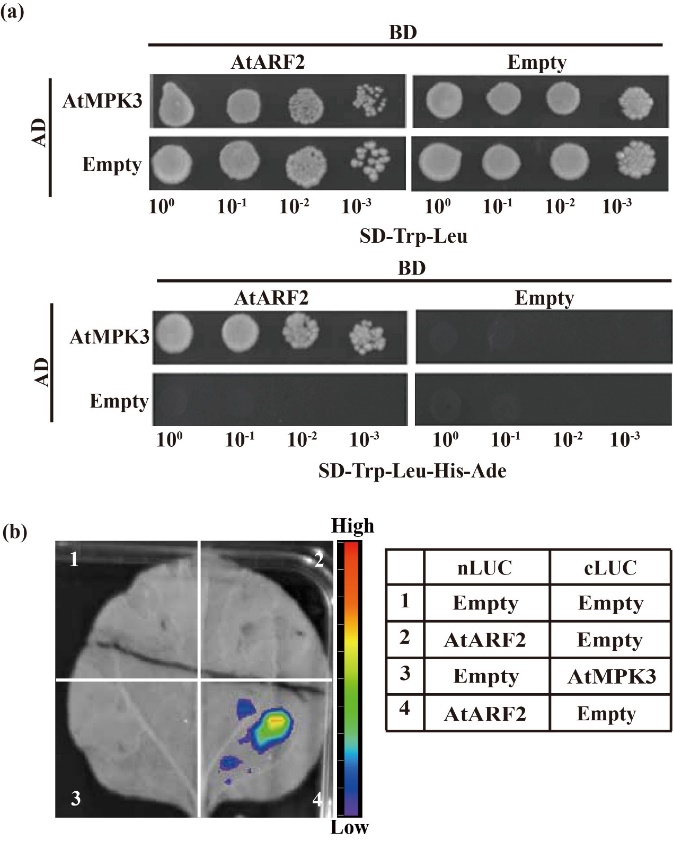


**Figure S7. AtMPK3 interacted with AtARF2.**

(a)AtMPK3 interacted with AtARF2 in the Y2H assay. AD, GAL4 activation domain fusion; BD, GAL4-binding domain fusion. BD-Empty/AD-Empty was used as negative control. Yeast cells were cultured on SD/-Trp-Leu or SD/-Trp-Leu-His-Ade media, and diluted as 10^0^, 10^-1^, 10^-2^ and 10^-3^. (b) AtMPK3 interacted with AtARF2 in the split luciferase complementary assay performed using the *N. benthamiana* leaves. nLUC, the vector containing the N-terminal fragment of firefly luciferase. cLUC, the vector containing the C-terminal fragment of firefly luciferase. AtARF2 was fused with nLUC and AtMPK3 was fused with cLUC. The *N. tabacum* leaves were infiltrated with the indicated combinations.


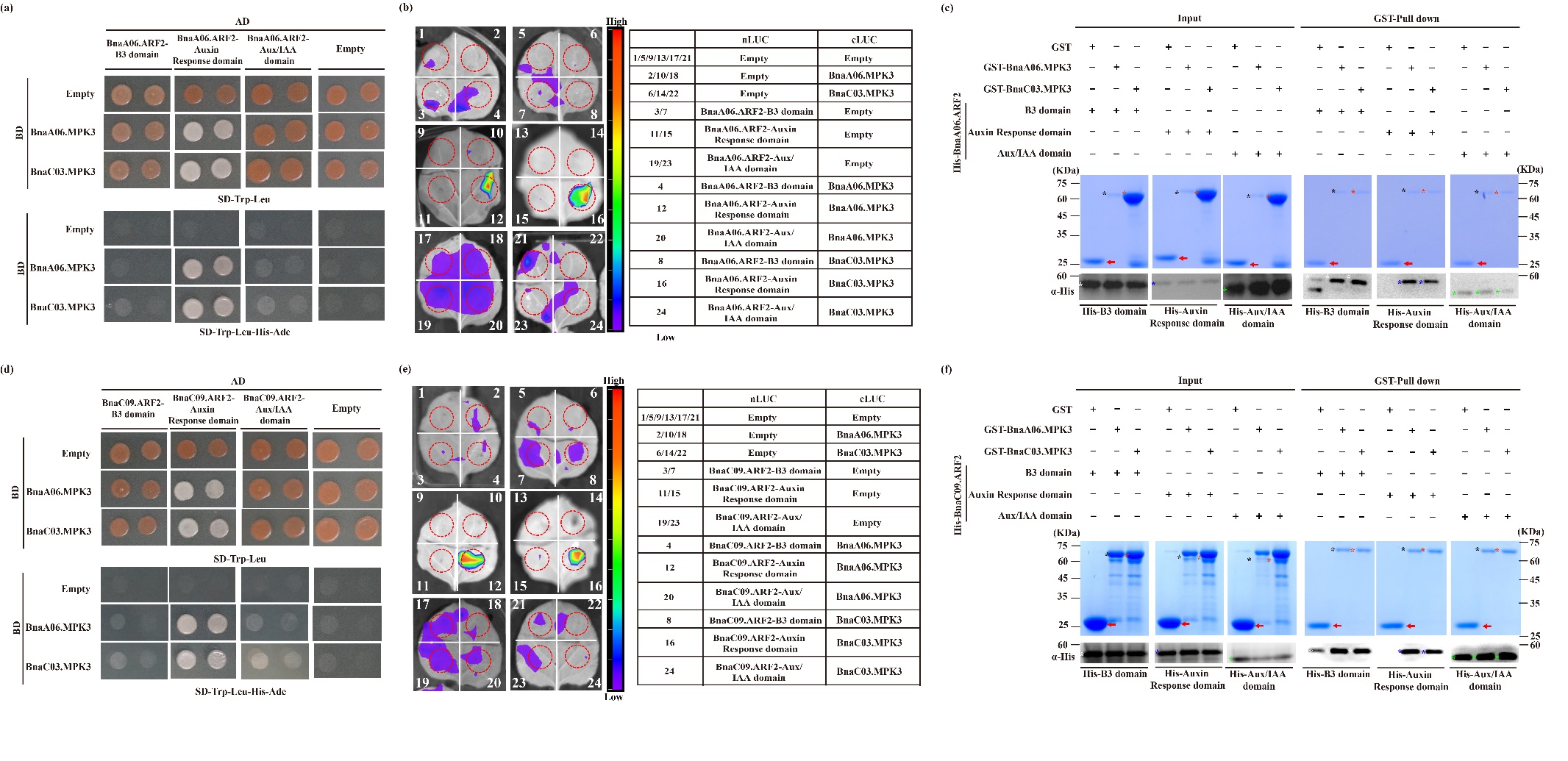
 **Figure S8. BnaMPK3s interacted with the Auxin Response domain of BnaARF2s.**

(a)-(c) BnaMPK3s interacted with the Auxin Response domain of BnaA06.ARF2 in the Y2H assay (a), split luciferase complementary assay in *N. benthamiana* (b), and pull-down assay (c). (d)-(e) BnaMPK3s interacted with the Auxin Response domain of BnaC09.ARF2 in the Y2H assay (d), split luciferase complementary assay in *N. benthamiana* (e), and pull-down assay (f). In (a)-(c), B3 domain: 1-285 AA; Auxin Response domain: 286-612 AA; Aux/IAA domain: 613-851 AA. In (d)-(f), B3 domain: 1-261 AA; Auxin Response domain: 262-586 AA; Aux/IAA domain: 587-828 AA. In (c) and (f), BnaMPK3s were fused with the GST-tag; truncated BnaA06.ARF2 or BnaC09.ARF2 was fused with the His-tag. The specific bands of GST were labelled by arrow; the specific bands of GST-BnaA06.MPK3, GST-BnaC03.MPK3 were labelled by black and red asterisk respectively. In (c), the specific bands of His-BnaA06.ARF2-B3, His-BnaA06.ARF2-Auxin Response and His-BnaA06.ARF2-Aux/IAA were labelled by white, blue and green asterisk, respectively. In (f), the specific bands of His-BnaC09.ARF2-B3, His-BnaC09.ARF2-Auxin Response and His-BnaC09.ARF2-Aux/IAA were labelled by white, blue and green asterisk, respectively.


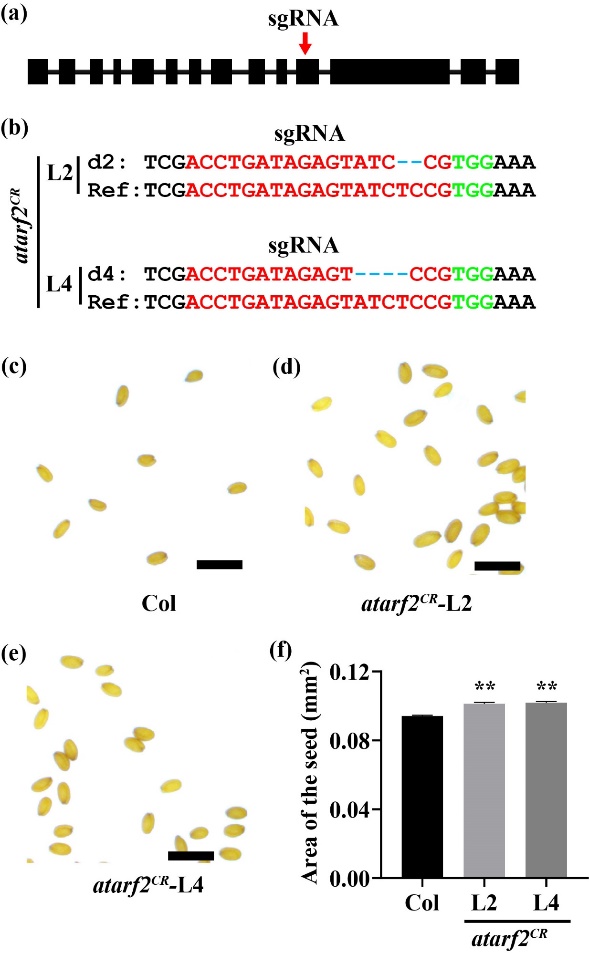


**Figure S9. *atarf2^CR^* increased the seed size.**

(a) The AtARF2 gene model included 14 exons (box) separated by 13 introns (represented by the solid line). The red arrow in the gene model indicated the sgRNA target site. (b) Genotype of the *atarf2^CR^* mutant in the T_1_ generation. The protospacer adjacent motif (PAM) region was marked in green. The sgRNA was denoted by red. The mutation sites were indicated with blue. d: deletion. (c)-(e) The images showed seed size of Col (c) and *atarf2^CR^* (d and e). Scale bars indicated 1 mm. (f) The bar graph showed the quantitative data from (c) to (e). The data were analyzed by Student’s t-test (*, P < 0.05; **, P < 0.01).


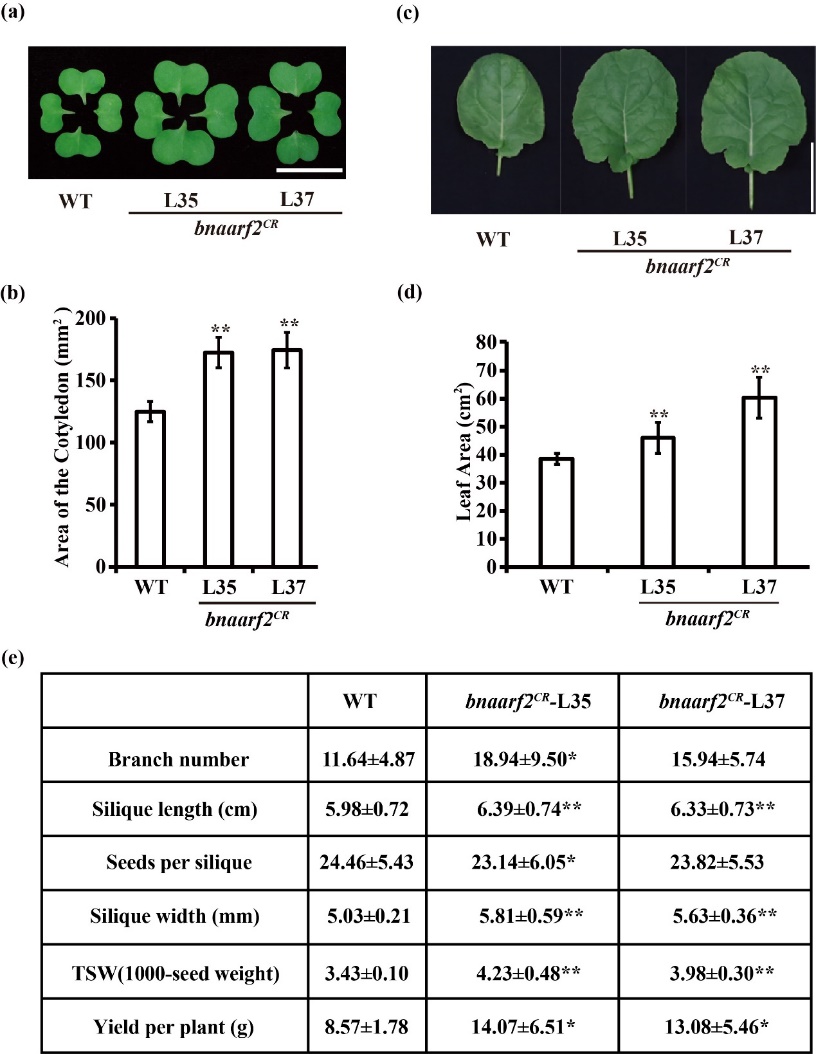


**Figure S10. The agronomic traits of *bnaarf2^CR^* and WT.**

(a)-(b) The image (a) and bar graph (b) showed the cotyledon size of 14-day-old *bnaarf2^CR^* and WT. In (a), scale bars indicated 20 mm. (c)-(d) The image (c) and bar graph (d) showed the leaf size of 35-day-old *bnaarf2^CR^* and WT. In (d), scale bars indicated 50 mm. (e) The table showed the yield related agronomic traits of *bnaarf2^CR^* and WT. Data were analyzed by Student’s t-test (*, P < 0.05; **, P < 0.01).


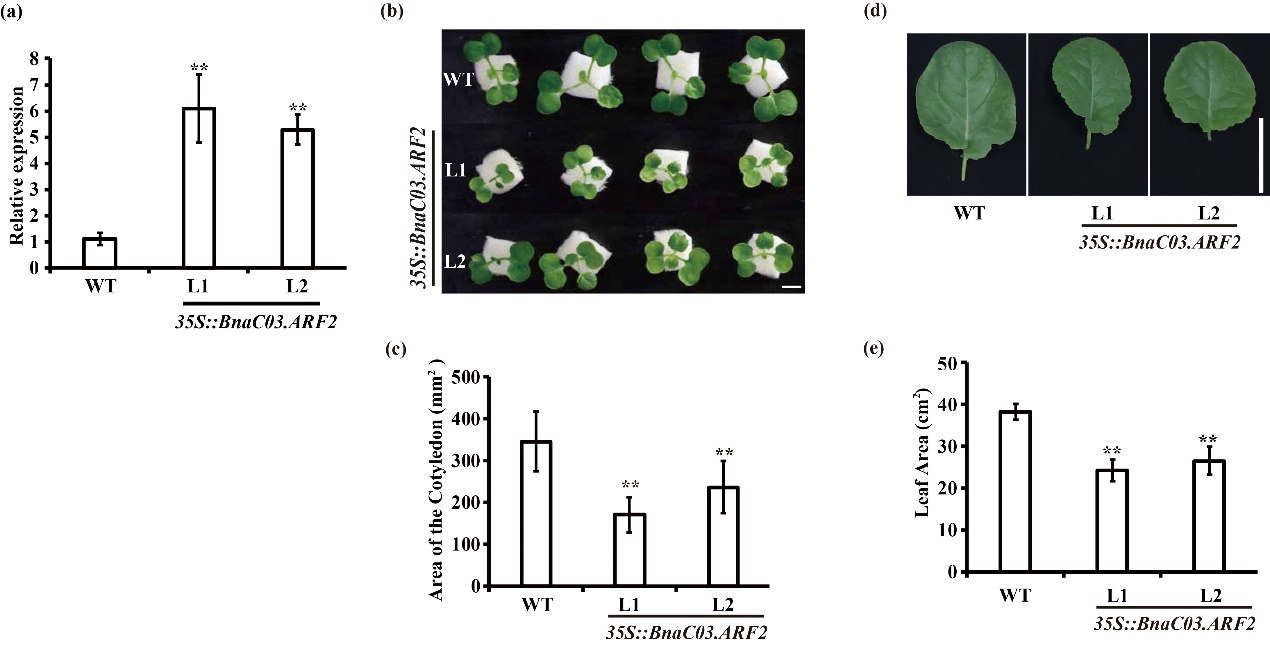


**Figure S11. The cotyledon and leaf size of *35S::BnaC03.ARF2* and WT.**

(a) Bar graph showed the relative expression levels of *BnaC03.ARF2* in homozygous *BnaC03.ARF2* overexpression transgenic lines and WT by quantitative real-time PCR. For *BnaC03.ARF2*, the expression level of WT was set as 1. *BnaActin* acted an internal control. (b)-(c) The image (b) and bar graph (c) showed the cotyledon size of 14-day-old *35S::BnaC03.ARF2* and WT. In (b), scale bars indicated 20 mm. (d)-(e) The image (d) and bar graph (e) showed the leaf size of 35-day-old *35S::BnaC03.ARF2* and WT. In (d), scale bars indicated 50 mm. Data were analyzed by Student’s t-test (*, P < 0.05; **, P < 0.01).


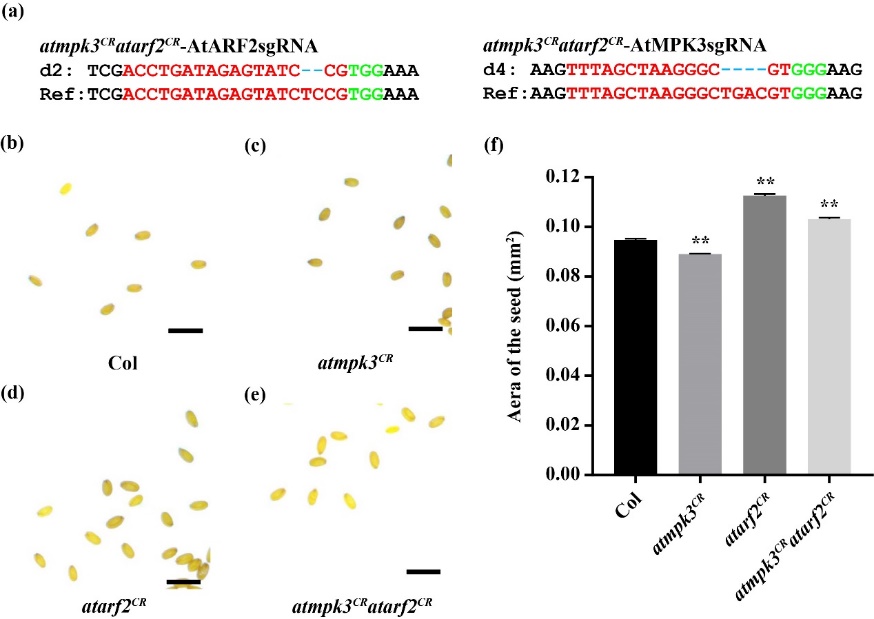


**Figure S12. ARF2 is epistatic to MPK3 in regulating seed size.**

(a) Genotype of the *atmpk3^CR^atarf2^CR^* mutant in the F_2_ generation. The protospacer adjacent motif (PAM) region was marked in green. The sgRNA was denoted by red. The mutation sites were indicated with blue. d: deletion. (b)-(e) The image showed the seed size of Col (b), *atmpk3^CR^* (c), *atarf2^CR^* (d), and *atmpk3^CR^atarf2^CR^* (e). Scale bars indicated 1 mm. (f) The bar graph showed the quantitative data from (b) to (e). The data were analyzed by Student’s t-test (*, P < 0.05; **, P < 0.01).


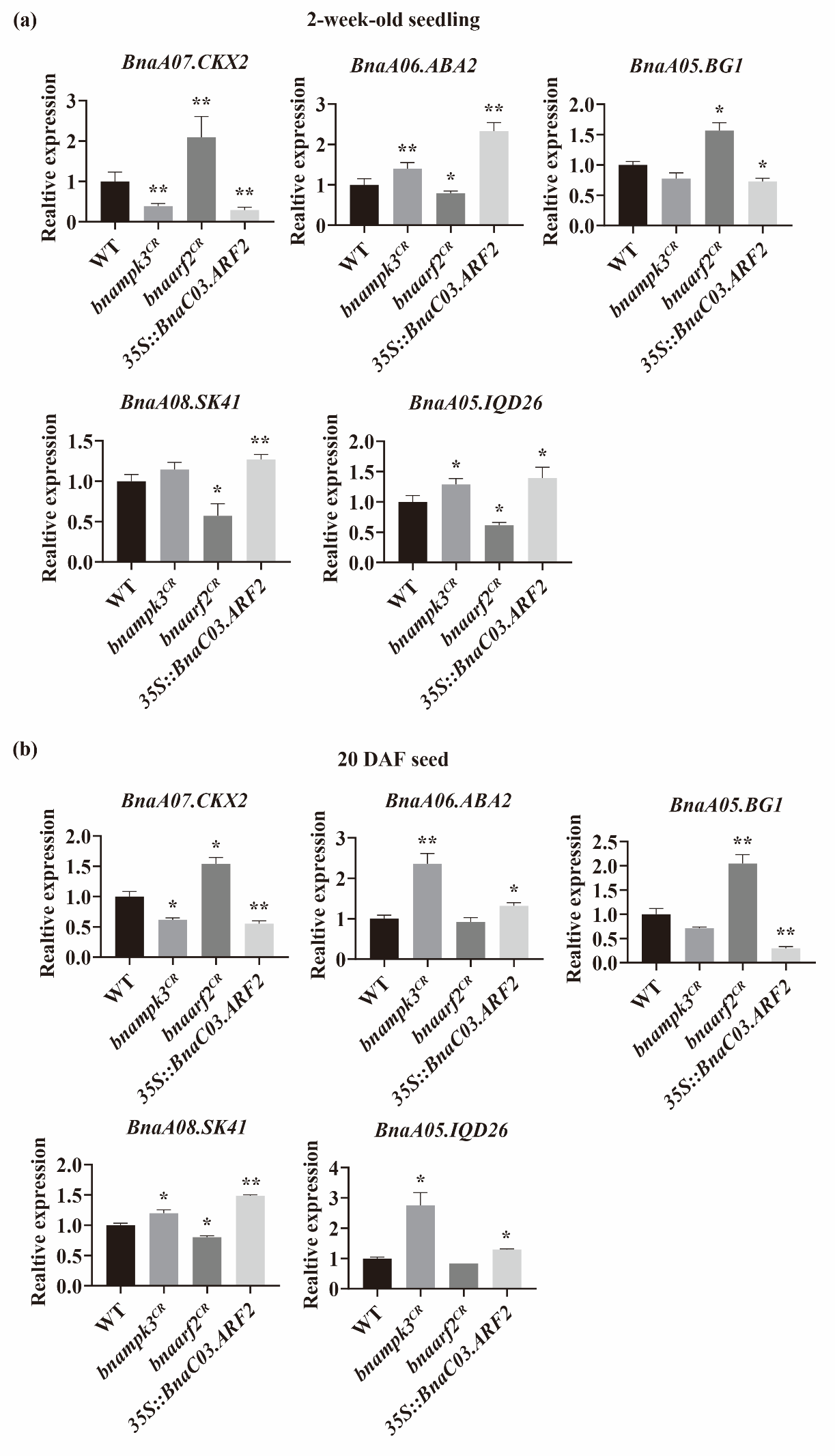


**Figure S13. The expression levels of selected DEGs in different materials.**

(a) The bar graph showed the expression levels of selected DEGs in 2-week-old seedlings of different materials. For each gene, the expression level of WT in 2-week-old seedling was set as 1. (b) The bar graph showed the expression levels of the selected DEGs in 20-day-after-flowering (DAF) seeds of different materials. For each gene, the expression level of WT in 20 DAF (day-after-flowering) was set as 1. In (a) and (b), *BnaActin* acted an internal control. Data were mean ± SE from three independent experiments, and analyzed by Student’s t-test (*, P < 0.05; **, P < 0.01).


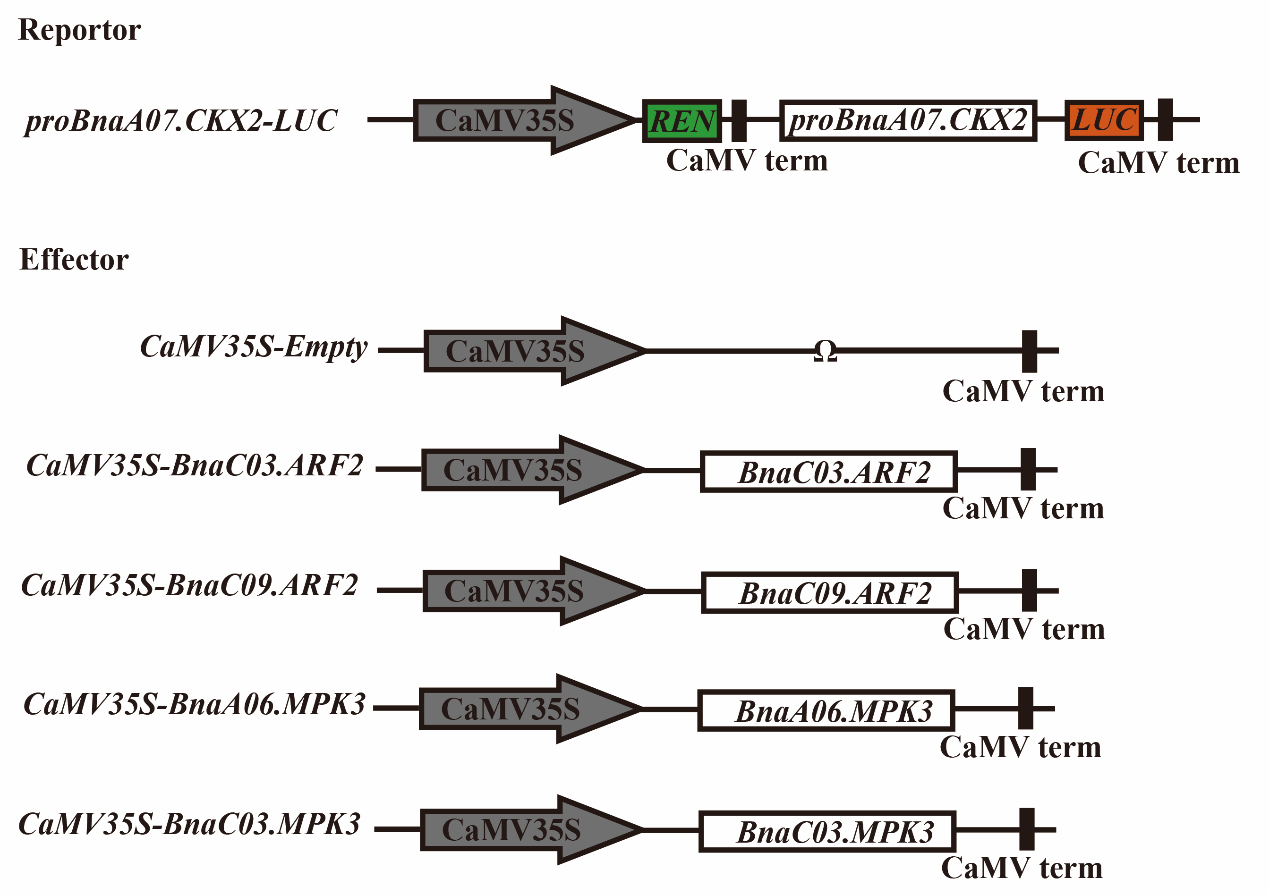


**Figure S14. The constructs used for the dual-luciferase assay.**

The reporter construct contained the firefly luciferase driven by *BnaA07.CKX2* promoter, and the Renilla luciferase (REN) was driven by the *CaMV 35S* promoter. The effector constructs contained BnaC03.ARF2, BnaC09.ARF2, BnaA06.MPK3 and BnaC03.MPK3 driven by the *CaMV 35S* promoter, respectively.


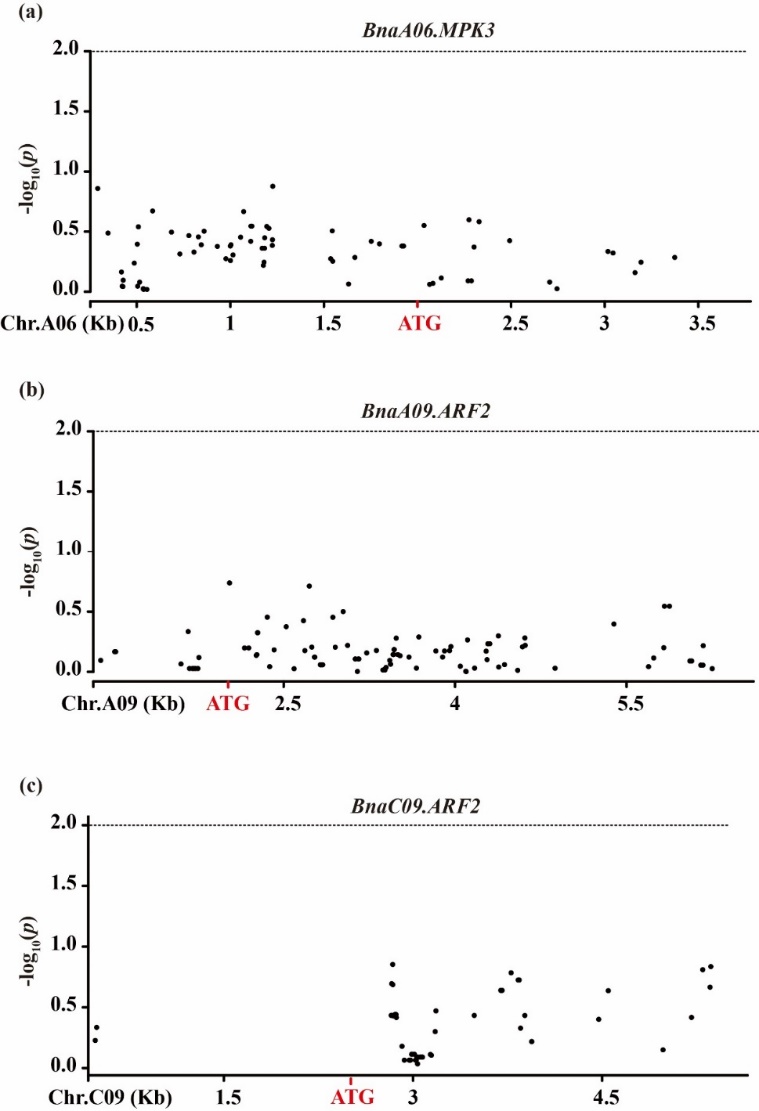


**Figure S15. Association analysis the *BnaA06.MPK3*, *BnaARF2s*.**

(a)-(c) Local Manhattan plot for *BnaA06.MPK3* (a), *BnaA09.ARF2* (b), and *BnaC09.ARF2* (c) of gene body and 2 kb upstream of the genes. The ATG indicated the transcription start site and transcription orientation. The dotted line was the significant threshold -log_10_(*p*) = -log_10_(0.01) = 2.0.


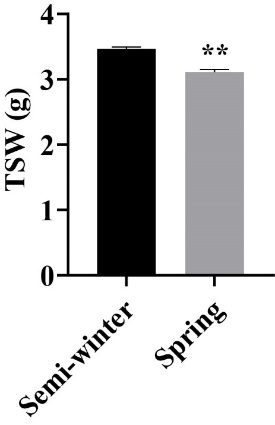


**Figure S16. The TSW of Spring and Semi-winter cultivars.**

The bar graph showed the TSW of Spring and Semi-winter cultivars in 505 *B. napus* natural population. Data were mean±SE. The data were analyzed by Student’s t-test (*, P < 0.05; **, P < 0.01).
